# JAK Inhibitors and Memory Impairment: Disproportionality Analyses in the WHO Global Pharmacovigilance Database, VigiBase

**DOI:** 10.1111/fcp.70072

**Published:** 2026-01-20

**Authors:** Marilou Duboëlle, Adriano Lercara, Yves‐Marie Pers, Céline Michel, Marion Lepelley, Marie‐Blanche Valnet‐Rabier, Jean‐Luc Faillie, Virginie Bres, Pascale Palassin

**Affiliations:** ^1^ Pharmacovigilance Regional Centre, Department of Medical Pharmacology and Toxicology CHU Montpellier Montpellier France; ^2^ IRMB, University of Montpellier, INSERM, Clinical Immunology and Osteoarticular Diseases Therapeutic Unit, Lapeyronie University Hospital CHU Montpellier Montpellier France; ^3^ Medical Centre Villeneuve‐de‐la‐Raho France; ^4^ Pharmacovigilance Regional Centre CHU Grenoble‐Alpes Grenoble France; ^5^ Pharmacovigilance Regional Centre, Department of Medical Pharmacology and Toxicology CHU Besançon Besançon France; ^6^ Pharmacovigilance Regional Centre, Department of Medical Pharmacology and Toxicology, CHU Montpellier, Desbrest Institute of Epidemiology and Public Health, Inserm Univ Montpellier Montpellier France

**Keywords:** adverse drug reaction, JAK inhibitor, memory impairment, pharmacovigilance

## Abstract

**Background:**

Chronic inflammation is involved in various mechanisms of memory impairment (MI). Although Janus kinase inhibitors (JAKi), which inhibit cytokine‐induced JAK–STAT pathway, could theoretically protect against MI, we faced an unexpected case of MI in a non‐elderly patient treated with JAKi.

**Objective:**

Our study aims to investigate the association between JAKi and MI.

**Methods:**

We searched VigiBase, the global pharmacovigilance database, for MI cases reported with JAKi from January 2011 to December 2023 and reviewed the literature for additional cases. The potential association was further explored through disproportionality analyses by calculating Reporting Odds Ratios (ROR), with statistical significance defined as a ROR and its 95% confidence interval exceeding 1.

**Results:**

A total of 3788 MI cases associated with JAKi were included, 36.3% of which were serious. Over half involved non‐elderly patients, and co‐reported confounding drugs were rare. According to disproportionality analyses, MI was reported nearly three times more frequently with JAKi than with all other drugs (ROR 2.92; 95% CI: 2.83–3.01). To illustrate, a 54‐year‐old woman with rheumatoid arthritis treated with tofacitinib for 6 months experienced MI with word‐finding difficulties (e.g., reduced categorical fluency: 25 animals named in 2 min; norm 30–47) and short‐term memory loss, fully resolved 6 weeks post‐discontinuation.

**Conclusion:**

Our data support the positive association between MI and JAKi, potentially mediated through hippocampal JAK/STAT pathway inhibition, impairing cholinergic neurotransmission and synaptic plasticity. While further investigations are warranted to confirm or refute this pharmacovigilance signal, clinicians should remain vigilant given this potentially serious adverse effect.

**Abbreviations**
ad
: Alzheimer's disease
ADR: adverse drug reaction
AE: adverse effect
ATC: Anatomical Therapeutic Chemical
BZD: benzodiazepines
CI: confidence interval
DMARD: disease‐modifying antirheumatic drug
EMA: European Medicines Agency
FDA: Food and Drug Administration
ICSR: individual case safety report
JAK: Janus kinase
JAK/STAT: Janus kinase/signal transducers and activators of transcription
JAKi: Janus kinase inhibitor
MACE: major adverse cardiovascular events
MedDRA: Medical Dictionary for Regulatory Activities
MI: memory impairment
MTX: methotrexate
PT: preferred term
RA: rheumatoid arthritis
ROR: reporting odds ratio
SD: standard deviation
STAT: signal transducers and activators of transcription
TNFαi: TNFα inhibitor
WHO: World Health Organization

## 1 Introduction

The origin of the name ‘JAK’ (Janus Kinase) is twofold; it refers first to the Roman god Janus, a two‐faced deity symbolizing duality—an allusion to the structural organization of these kinases. Second, ‘JAK’ also stood for ‘Just Another Kinase’, highlighting early uncertainty about their function and hinting at their pleiotropic role in diverse signalling pathways [1]. The JAK/STAT pathway (Janus kinase/signal transducers and activators of transcription) regulates key physiological processes, such as cell proliferation and differentiation and plays an essential role for transmitting pro‐inflammatory and immune signals [2]. In the 2010s, Janus kinase inhibitors (JAKi) emerged as a promising class of drugs for the treatment of haematological diseases and various autoimmune or inflammatory conditions (e.g., rheumatoid arthritis [RA], chronic inflammatory bowel diseases and atopic dermatitis) [3, 4]. These drugs act by inhibiting intracellular Janus kinases (JAK1, JAK2, JAK3 and TYK2), with each molecule exhibiting distinct selectivity profiles towards specific JAK isoforms.

In addition to its effects on the immune and inflammatory systems, the JAK/STAT pathway also impacts the central nervous system, particularly glial and neuronal cells, regulating essential neurological processes such as synaptic plasticity [5, 6] and neurogenesis [6, 7], both crucial for maintaining cognitive functions, including memory. Conflicting hypotheses remain about the impact of JAKi on the brain: Some suggest neuroprotection via reduced cerebral inflammation [8, 9, 10, 11], while others propose adverse effects (AEs) on cognition, particularly memory [7, 12, 13, 14, 15].

JAKi are associated with various AEs [1, 16, 17]. Due to their use in chronic diseases, prolonged exposure raises concerns about long‐term safety. In addition to well‐established class‐related risks, concerns have recently emerged. Notably, the ORAL Surveillance study evidenced a higher risk of major adverse cardiovascular events (MACE) and cancers (excluding non‐melanoma skin cancers) with tofacitinib, a JAKi, compared to TNFα inhibitors (TNFαi) [18], prompting the European Medicines Agency (EMA) to implement risk‐reduction measures [19]. Another recent study showed that JAKi were more associated with MACE, especially stroke [20]. However, cognitive disorders, particularly memory impairment (MI), remain under‐investigated and poorly documented in the literature. This study was initiated following an unexpected case of MI during tofacitinib treatment, with a highly suggestive temporal association. To date, no clear pharmacovigilance signal has been identified, and to our knowledge, this is the first study specifically designed to assess the association of MI with JAKi. Our primary objective was to investigate a potential signal of MI associated with JAKi using the World Health Organization's (WHO) global pharmacovigilance database. Second, mechanistic hypotheses were explored from a literature review. This study, therefore, seeks to identify a class effect of JAKi on cognitive disorders and contribute to a more comprehensive safety profile for this therapeutic class.

## 2 Materials and Methods

### 2.1 Data Source

This study is a retrospective pharmacovigilance analysis, which does not require prior registration in a public registry. Data were obtained from VigiBase, the WHO global database of individual case safety reports (ICSRs), with access granted by the Uppsala Monitoring Centre (UMC) to authorized experts under confidentiality agreements. All data were extracted from this database from 1 January 2011, the year of the first commercialization of JAKi, to 31 December 2023. VigiBase is the largest pharmacovigilance database, collecting reports from over 160 member countries, with more than 40 million ICSRs submitted by pharmaceutical manufacturers, healthcare professionals and consumers through national pharmacovigilance systems [21]. Each case, also known as an ICSR, contains structured data on factors such as sex, age, country of origin, suspected adverse reactions and implicated drugs. All adverse drug reactions (ADRs) are coded according to the Medical Dictionary for Regulatory Activities (MedDRA), version 26.1. A case is considered serious if it meets at least one of the following criteria: death, life threatening, caused/prolonged hospitalization, disabling/incapacitating, congenital anomaly/birth defect and other medically important condition. The final criterion depends on medical expert opinion.

### 2.2 Exposure and Outcome Definitions

To account for the fact that JAKi are widely dispersed across multiple Anatomical Therapeutic Chemical (ATC) classes and are approved for a broad range of therapeutic indications—including dermatologic, rheumatologic, gastrointestinal and haematologic diseases—we created a custom drug group comprising all JAKi listed in the database (*n* = 20): abrocitinib, baricitinib, brepocitinib, cerdulatinib, decernotinib, delgocitinib, deucravacitinib, fedratinib, filgotinib, gandotinib, gusacitinib, itacitinib, momelotinib, pacritinib, peficitinib, ritlecitinib, ropsacitinib, ruxolitinib, tofacitinib and upadacitinib.

We defined a custom reaction group in VigiBase based on the semiological entity corresponding to MI. We selected de‐duplicated ICSRs within the MedDRA High Level Term ‘Memory loss (excl dementia)’ in which we excluded all the MedDRA Preferred Terms (PTs) related to neurodegenerative, deficiencies or post‐traumatic disorders, assuming that JAKi are unlikely to cause reversible MI through such mechanisms. The final outcome group included six PTs: ‘Amnesia’, ‘Amnestic disorder’, ‘Anterograde amnesia’, ‘Memory impairment’, ‘Retrograde amnesia’ and ‘Transient global amnesia’.

We extracted all ICSRs reporting at least one of the six selected PTs in association with any of the 20 JAKi classified as either suspect or interacting. In the final step, a case‐by‐case manual review was conducted, and cases with co‐reported PTs classified under investigator‐defined categories—vascular disorders, degenerative disorders, brain disorders and brain injuries—were excluded (Table S1).

### 2.3 Data Analysis

Standard descriptive statistics used proportions, means and standard deviations (SDs).

We performed a disproportionality analysis based on ICSRs using the case/non‐case method to identify disproportionate reporting, meaning a higher‐than‐expected number of adverse reaction reports compared to other reactions recorded in the database [21]. Reporting odds ratios (RORs) with 95% confidence intervals (CI) were calculated. Cases were defined as all reports involving MI, and non‐cases included all other ADR reports in the same period. Exposure to JAKi was compared between cases and non‐cases to all other drugs listed in this database, regardless of their active ingredient and indication. A signal of disproportionate reporting was considered statistically significant if the lower bound of the 95% CI for the ROR exceeded 1 and at least three drug‐event combinations were observed [22].

Several complementary analyses were performed to further explore this potential association. Disease‐modifying antirheumatic drugs (DMARDs) were selected using the standardized drug groups ‘SDG – Narrow: Disease‐modifying antirheumatic drugs (DMARDs)’. Given the lack of a single optimal comparator, we used multiple comparators to address distinct questions. Methotrexate (MTX) was included for its shared rheumatologic and haematologic indications with JAKi, while TNFαi were added as standard comparators in previous studies (e.g., ORAL Surveillance) and for their alignment with advanced disease stages. Accordingly, we compared the four JAKi approved for rheumatologic conditions (tofacitinib, baricitinib, upadacitinib, filgotinib) with DMARDs (Figure 2), as patients with rheumatologic diseases represented the majority of our cohort. As a positive control, we checked the association with drugs known to be related to MI such as benzodiazepines (BZD) and Z‐drugs (N05CD and N05CF ATC classes). As a negative control, we assessed the association with paracetamol, not known to be associated with cognitive AEs.

We followed the READUS‐PV guidelines for reporting disproportionality analyses [23].

### 2.4 Literature Review

A literature review was performed in the Medline biomedical database to identify similar case reports using MeSH terms as of 30 March 2024: (‘Neurologic Manifestations’[Mesh]) AND (‘Janus Kinases’[Mesh]), without applying any time restrictions. Articles were first screened by title, followed by abstract review if the title alone did not clarify relevance to our search criteria. Based on the results of this review, we then conducted a recursive bibliographic search to gain a deeper understanding of the underlying pharmacological mechanisms.

## 3 Results

### 3.1 Pharmacovigilance Data Analyses

**FIGURE 1:**
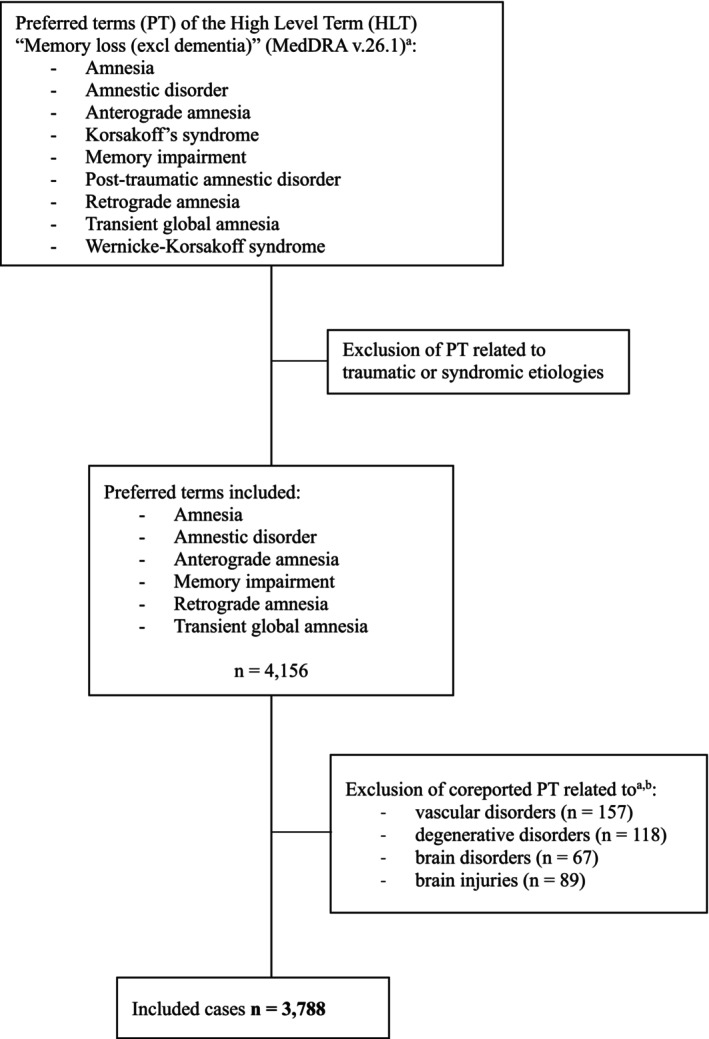
Flow diagram of the selection of outcome terms. ^a^One ICSR corresponds to one patient. A patient may have experienced one or more adverse events. ^b^Table S1 details of the excluded co‐reported PTs. HLT: High Level Term of the MedDRA classification; MedDRA: Medical Dictionary for Regulatory Activities; PT: Preferred Term of the MedDRA classification.

A total of 3788 ICSRs of MI with at least one JAKi as suspect drug were selected from the WHO pharmacovigilance database, after applying the exclusion criteria (Figure 1). Table 1 summarizes the main characteristics of these reports. Among the 20 JAKi included in the search, only eight (40.0%) were implicated, and they were the single suspected drug in 91.6% of cases. Tofacitinib was the most frequently reported (*n* = 2632; 69.5%), followed by ruxolitinib (*n* = 858; 22.7%), with the remaining six other JAKi accounting for almost 8.0% of the cases. There were only two cases in which two different JAKi were suspected. Interestingly, women represented the majority of patients (80.9%), and 57.1% were under 65 years old. Most reports were submitted by consumers or non‐healthcare professionals (60.2%), with a predominance of cases originating from the United States (87.2%). The highest annual reporting rate was observed in 2022 (21.6%). Rheumatic disorders were the most frequently mentioned indications (66.0%), especially RA. Overall, 36.3% of MI cases were considered serious, primarily due to being a medically important condition (88.9%). Patients were taking an average of 3.5 medications (SD 5.5), with 81.0% reporting between one and five drugs.

**TABLE 1:** Characteristics of MI cases reported with JAKi treatment in the WHO pharmacovigilance database, VigiBase.

Characteristics	Cases (*n* = 3788)
Age (years), *n* (%)	2828
Median (P25–P75)	62 (52–71)
< 18	9 (0.3)
18–49	526 (18.6)
50–64	1081 (38.2)
≥ 65	1212 (42.9)
Unknown	960
Gender, *n* (%)	3103
Male	594 (19.1)
Female	2509 (80.9)
Unknown	685
Reporter type^a^, *n* (%)	3774
Health professional	1501 (39.8)
Non‐health professional	2273 (60.2)
Unknown	14
Reporting year, *n* (%)	3788
2013	1 (0.0)
2014	32 (0.8)
2015	80 (2.1)
2016	84 (2.2)
2017	200 (5.3)
2018	357 (9.4)
2019	517 (13.6)
2020	542 (14.3)
2021	650 (17.2)
2022	818 (21.6)
2023	507 (13.4)
Reporting countries, *n* (%)	3788
USA	3302 (87.2)
Others (24 countries)	486 (12.8)
Cases including PT of interest^b^, *n* (%)	3788
Memory impairment	3353 (88.5)
Amnesia	479 (12.6)
Transient global amnesia	9 (0.2)
Retrograde amnesia	0 (0.0)
Anterograde amnesia	0 (0.0)
Amnestic disorder	0 (0.0)
Cases with at least one suspected JAKi, *n* (%)	3788
Tofacitinib	2632 (69.5)
Ruxolitinib	858 (22.7)
Upadacitinib	208 (5.5)
Baricitinib	64 (1.7)
Pacritinib	12 (0.3)
Fedratinib	9 (0.2)
Abrocitinib	6 (0.2)
Deucravacitinib	1 (0.0)
Main indications of JAKi, *n* (%)	2750
Rheumatic disorders	1814 (66.0)
Rheumatoid arthritis	*1691 (93.2)*
Onco‐haematological disorders	757 (27.5)
Myelofibrosis and polycythaemia vera	*633 (83.6)*
Autoimmune or inflammatory diseases	155 (5.6)
Colitis	*123 (81.3)*
Others	24 (0.9)
Unknown	1038
Cases with co‐reported BZD and Z‐drugs, *n* (%)	161 (4.3)
Suspect/interacting	8 (5.0)
Concomitant	153 (95.0)
Cases with co‐reported MTX, *n* (%)	296 (7.8)
Suspect/interacting	79 (26.7)
Concomitant	217 (73.3)
Time to onset, *n* (%)	89 (2.4)
Median (days) (P25–P75)	395.2 (152.0–1095.8)
< 1 month after initiation	7 (7.9)
≥ 1 month and < 6 months after initiation	18 (20.2)
≥ 6 months after initiation	64 (71.9)
Unknown	3699
Dechallenge performed, *n* (%)	780 (20.6)
Unknown	3008
Result of dechallenge	
Resolved/resolving reaction	73 (9.4)
Unresolved reaction	116 (14.9)
Unknown	591 (75.8)
Rechallenge performed, *n* (%)	484 (12.8)
Unknown	3304
Result of rechallenge	
Recurred reaction	2 (0.4)
Non‐recurred reaction	9 (1.9)
Unknown	473 (97.7)
Severity of cases, *n* (%)	3786
Serious cases^c^	1375 (36.3)
Seriousness criteria, *n* (%)	
Other medically important condition	1223 (88.9)
Caused/prolonged hospitalization	443 (32.2)
Disabling/incapacitating	57 (4.1)
Death	44 (3.2)
Life threatening	29 (2.1)
Non‐serious cases	2411 (63.7)
Unknown	2
Evolution, *n* (%)	730 (19.3)
Recovered/recovering	202 (27.7)
Not recovered	522 (71.5)
Died	5 (0.8)
Unknown	3060

*Note:* Percentages were calculated based on available data.

Abbreviations: BZD: benzodiazepines; JAKi: JAK inhibitors; MTX: methotrexate.

^a^ Cases reported by several reporter types, where at least one was a health care professional, were considered as reported by a health care professional.

^b^ Preferred term of the MedDRA classification.

^c^ Cases that include at least one seriousness criterion as specified by pharmacovigilance regulations.

**FIGURE 2:**
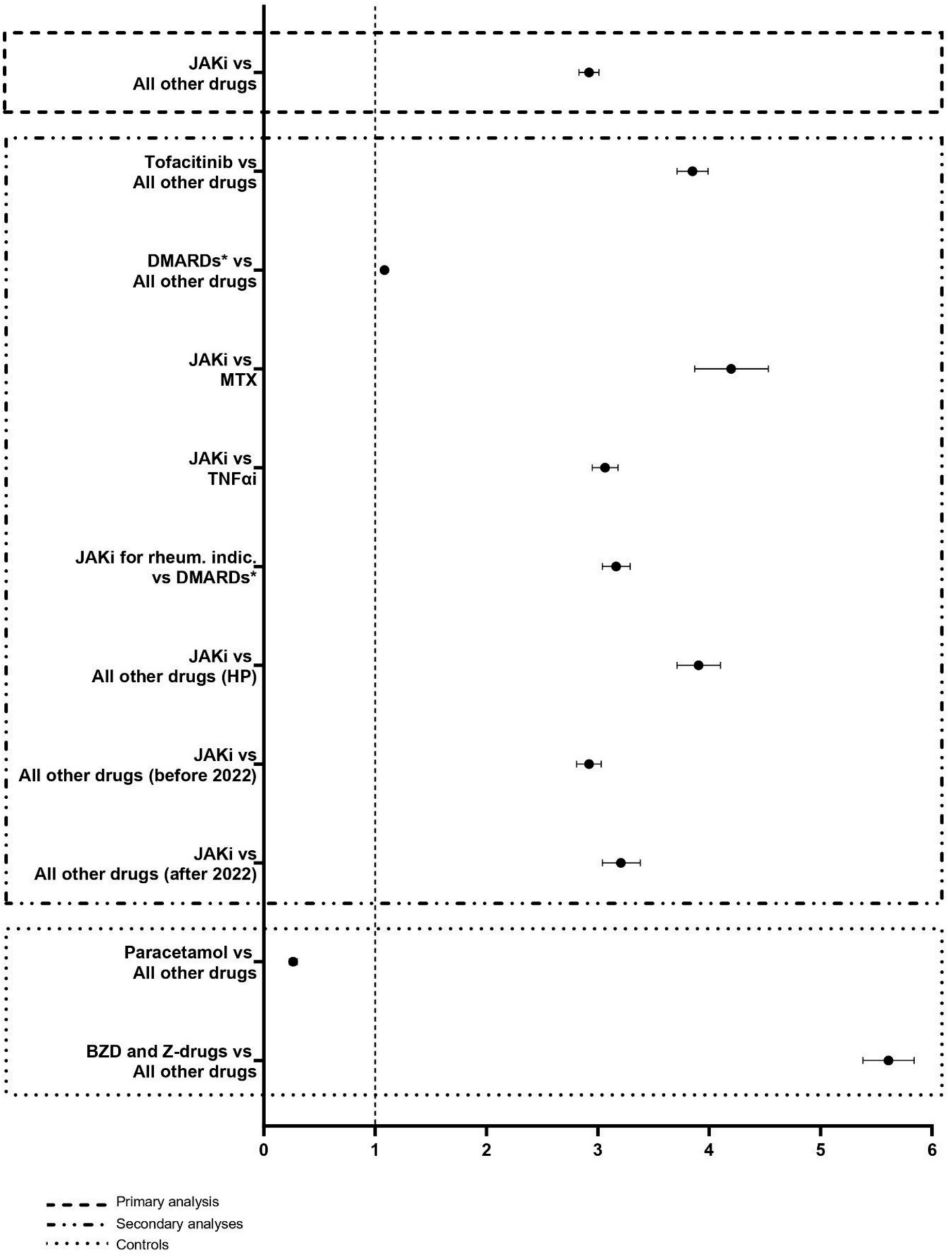
Disproportionality analyses of MI reports (reporting odds ratios) based on VigiBase data. Forest‐plot illustrating disproportionality analyses of MI cases. *DMARDs excluding tofacitinib, the only JAKi classified as a DMARD. JAKi for rheumatologic indications: tofacitinib, upadacitinib, baricitinib and filgotinib. BZD: benzodiazepines; DMARDs: disease‐modifying antirheumatic drugs; HP: healthcare professional; JAKi: JAK inhibitors; MTX: methotrexate; TNFαi: TNFα inhibitors.

Figure 2 presents the results of disproportionality analyses. MI was nearly three times more reported with JAKi than with all other drugs (ROR 2.92; 95% CI: 2.83–3.02), with the strongest signal observed for tofacitinib (ROR 3.85, 95% CI: 3.71–3.99). This signal remained regardless of the underlying rheumatic condition, as MI was reported more than three times as often with the four JAKi indicated for rheumatic conditions (tofacitinib, baricitinib, upadacitinib and filgotinib) compared to other DMARDs (ROR 3.16; 95% CI 3.04–3.29). Similarly, the ROR remains significant and increases further when only reports made by healthcare professionals are considered (ROR_JAKi vs all drugs_ 3.90; 95% CI: 3.71–4.10).

### 3.2 Valuable Insights From a Clinical Study

To illustrate, we use the example of a 54‐year‐old woman, whose comorbidities included hypothyroidism and RA, who had been treated with levothyroxine sodium (Levothyrox) 50 μg and MTX 10 mg once weekly since 2017. Tofacitinib (Xeljanz) was initiated in June 2020 at a dose of 5 mg twice a day orally.

In December 2020, approximately 6 months after the initiation of tofacitinib, she developed memory disturbances characterized by word‐finding difficulties, word inversion and short‐term memory loss. In March 2021, because of a suspected AE of tofacitinib, the dosage was reduced to 5 mg per day. The cognitive symptoms gradually improved but did not fully resolve. A cognitive assessment performed in September 2021 was normal. However, there was a discrepancy in verbal fluency. Categorical verbal fluency was significantly reduced (the patient could name only 25 animals in 2 min; expected range of 30–47), whereas phonemic verbal fluency was preserved (she could name 27 words beginning with the letter ‘P’ in 2 min; expected ≥ 25). Additional investigations, including brain magnetic resonance imaging and Doppler ultrasound of the supra‐aortic trunks, revealed no abnormalities suggestive of neurodegenerative or vascular disorders. Tofacitinib was stopped in October 2021 due to RA remission and suspected AE with progressive and complete resolution 2 months later. The positive dechallenge was corroborated by normalization of memory tests, including above‐average verbal fluency. Neurological assessment concluded that there were no signs of early neurodegenerative disease. This case was registered in the global pharmacovigilance database under the identification number FR‐AFSSAPS‐MP20217609. The patient gave her consent for publication.

### 3.3 Literature Review

The literature search identified 152 publications, but none reported a similar case of MI associated with JAKi.

## 4 Discussion

### 4.1 Summary of Findings and Interpretation

To our knowledge, we report the first case illustrating the potential association between MI and JAKi. The compelling clinical case, marked by a suggestive chronology, prompted us to investigate this potential association further. The young patient age, the rapid and complete remission after drug withdrawal and the apparent dose‐dependency support a neurotoxicological rather than neurodegenerative mechanism. These considerations justified a comprehensive pharmacovigilance analysis of JAKi‐related MI cases from the WHO pharmacovigilance database. Most cases originated from the United States, plausibly reflecting the early market authorization of JAKi in this country. With 3788 reported cases, our pharmacovigilance study provides a reliable description of a poorly recognized concern. With the expansion of JAKi indications and the increasing number of patients treated, reports of MI have steadily risen since 2013 with a notable surge in 2022. Following the findings of the ORAL Surveillance study, both the Food and Drug Administration (FDA) and EMA issued warnings regarding serious adverse events associated with JAKi use, including cardiovascular events, thrombosis, malignancy and infections [18, 19, 24]. This has raised the possibility of a notoriety bias influencing the reporting rates [25]. Secondary analyses performed pre‐ and post‐2022 to evaluate the impact of safety alerts revealed no substantial differences (Figure 2). MI cases were considered serious in 36.3% of cases, implying a meaningful impact on quality of life.

In line with its widespread use, tofacitinib was involved in 69.5% of the cases. The marked female predominance and median age of the patients reporting MI were consistent with the epidemiology of RA [26]. Interestingly, a majority of cases involved patients under 65 years of age, which is unexpected in MI. As commonly observed in pharmacovigilance databases, time‐to‐onset data were largely missing. The limited available data suggest onset typically occurred beyond 6 months of treatment, compatible with a cumulative dose‐dependent toxic mechanism. This aligns with the pharmacokinetics of tofacitinib (maximum plasma concentrations are reached in 0.5 to 1 h and steady‐state concentrations in 24–48 h) [27]. Concomitant medications known to cause MI, such as BZD and Z‐drugs, did not appear to be a major confounding factor, given the low proportion of cases concerned. Pharmacovigilance data typically reflect a single time‐point report, with follow‐up information often missing due to limited hindsight at the time of reporting and lack of subsequent updates. Positive dechallenges can be underestimated, while negative dechallenges can be overestimated.

Compared to all other drugs, JAKi are associated with a significantly higher reporting of MI, but in contrast, DMARDs overall show no increased risk. The signal remains strong when comparing JAKi directly with MTX. It also remains elevated when compared with TNFαi, which are commonly used as JAKi comparators due to their overlapping indications. Overall, these results suggest a consistent and notably higher reporting of memory‐related adverse events with JAKi compared to other treatments commonly used in similar patient populations.

Paracetamol, as a negative control, is not associated with MI, as expected. BZD and Z‐drugs, known to cause amnesia, showed a strong link. Although a causal relationship between MI and JAKi could not be definitively established, our disproportionality analyses suggested that JAKi may increase the risk of MI.

### 4.2 Mechanistic Hypotheses

The use of JAKi for the treatment of inflammatory conditions could raise the question of confounding by indication, namely, whether MI could be related to the underlying inflammatory diseases rather than to JAKi exposure. However, secondary analyses indicated that the use of JAKi was associated with an even greater frequency of MI compared to MTX, thereby supporting the involvement of JAKi. In addition, given the previously identified safety signals, it is conceivable that JAKi‐associated thromboembolic events, such as strokes, could have resulted in vascular‐related MI. However, our exclusion criteria substantially minimize this possibility.

The JAK/STAT pathway could be critical in memory, but available studies remain limited and conflicting. Cognitive impairment has been reported in chronic systemic inflammatory diseases, particularly RA [28]. The main pathophysiological mechanisms involve persistent systemic inflammation, contributing to accelerated atherosclerosis and autoantibodies production [9]. JAKi should then have a potential neuroprotective effect through the reduction of neuroinflammation. However, some hypotheses may support a mechanistic link between JAK/STAT pathway inhibition and MI, a process commonly associated with ageing, Alzheimer's disease (ad), brain injuries or stroke [29]. The underlying mechanisms may vary according to aetiology. Although our study did not focus on neurodegenerative or irreversible processes, experimental findings on the JAK/STAT pathway provide mechanistic insights. In models of ageing and ad, irreversible β‐amyloid peptide accumulation has been shown to inhibit the JAK2/STAT3 pathway in hippocampal neurons [12, 14]. This inhibition would lead to desensitization of M(1)‐type muscarinic acetylcholine receptors and reduce levels of acetylcholine and cholinergic enzymes such as choline acetyltransferase and acetylcholinesterase, thereby disrupting cholinergic transmission [12, 13], a known contributor to MI. Neuroprotective agents such as humanin or colivelin can activate the JAK2/STAT3 pathway and mitigate these effects. However, experimental JAKi compounds (AG43 and AG490) appear to reverse this protective effect, exacerbating cognitive impairment [14]. Moreover, the JAK/STAT pathway could improve synaptic plasticity through STAT3 activation in the hippocampus [5, 6]. This pathway inhibition could then reduce neurogenesis and affect astrocyte proliferation, both crucial for memory [7]. These findings suggest that inhibiting the JAK/STAT pathway, particularly JAK2/STAT3, may be involved in impaired memory.

The complexity of JAK/STAT signalling pathways lies in their diversity and in the variability of biological responses depending on the degree of inhibition of each individual pathway. MI reported with tofacitinib accounts for more than two‐thirds of cases, likely due to its widespread use. This drug functions as a pan‐JAK inhibitor, with predominant activity on JAK1 and JAK3 pathways. Investigating a potential correlation between MI and the selectivity of JAKi would be of interest. However, the lack of available JAKi with exclusive specificity for a single JAK isoform limits the ability to perform a differential analysis of the observed effects. Experimental studies are warranted to elucidate the specific consequences of inhibiting each individual JAK/STAT pathway.

### 4.3 Strengths and Limits

Given the paucity of data in the literature, our study provided a comprehensive collection of available cases of MI reported with JAKi worldwide. As VigiBase access is restricted to authorized experts under confidentiality agreements, independent replication of these analyses by external researchers may be limited. Pharmacovigilance studies remain an essential tool for detecting new safety signals, monitoring AEs in real‐world settings and thus ensuring the safe use of medications. Nevertheless, this study presents limitations inherent to retrospective pharmacovigilance studies including the lack of established causality, underreporting, varying reporting practices across countries or potential notification biases such as the tendency to primarily report serious AEs or those related to new drugs [30]. Another limitation of VigiBase analysis is missing data, such as past medical history, complete work‐up and management strategies. Outcomes are frequently undocumented in the international ICSRs, partly explained by early reporting, the reporter type and the lack of follow‐up. In our study, information on dechallenge and its outcome, key elements for chronological evaluation, was largely missing, representing an important limitation. This is one of the reasons why the WHO categorization of JAK inhibitors as ‘suspect’ or ‘interacting’ does not ensure causality, and misclassification cannot be excluded. Media, public, or scientific attention may also influence reporting through notoriety effect [25]. The contrast between the large number of cases reported in the international pharmacovigilance database and the absence of cases in the literature is striking. This discrepancy raises concerns about the validity of diagnoses, especially since most reports were submitted by patients themselves. While one‐third of global VigiBase ICSRs are reported by non‐health professionals, this feature rose to 60.2% in our dataset. This predominance of patient reporters may have introduced a limitation by emphasizing patient‐reported symptoms rather than medically validated diagnoses. Nevertheless, this bias did not seem to impact the ROR values, which are reinforced when considering only healthcare professionals' reports. Consistent with our study objectives, the analysis focused on investigating a potential class effect associated with the shared mechanism of action of JAK inhibitors. However, it is also important to acknowledge certain methodological constraints and a posteriori stratification by indication was not feasible related to our dataset structure, which could represent a significant limitation. Therefore, a reported case cannot be considered as a confirmed ADR, nor can the reporting rate be interpreted as its actual incidence, nor disproportionality analyses a measure of real risks. A safety signal is information suggesting a potentially causal association that warrants further investigations. Therefore, the signal identified in this study should be interpreted as a hypothesis requiring additional confirmatory research [31]. However, in many examples, disproportionate reporting has proven to be correlated with identified drug‐related risks [32]. Despite these limitations, our analysis combining a well‐documented index case with the largest dataset of JAKi‐associated MI strongly supports the likelihood of JAKi‐induced MI.

## 5 Conclusion

This study combines a compelling clinical case with a large international dataset, disproportionality analyses and mechanistic hypotheses to explore a potential link between the JAK/STAT pathway inhibition and MI. Our findings highlight the importance of monitoring cognitive effects among patients treated with JAKi. Further research, including pharmaco‐epidemiological studies, is needed to confirm and better assess the impact of JAK/STAT inhibition on cognitive function, and refine the benefit–risk assessment of JAKi.

## Author Contributions

Marilou Duboëlle contributed to the acquisition and interpretation of data, drafting and revision of the manuscript. Pascale Palassin and Virginie Bres equally contributed to the design of the study, the acquisition and interpretation of data, drafting, revision and approval of the manuscript. Adriano Lercara, Yves‐Marie Pers, Marie‐Blanche Valnet‐Rabier, Marion Lepelley and Jean‐Luc Faillie contributed to revision, critique and approval of the manuscript. Céline Michel reported the clinical case to the pharmacovigilance system. All authors agree to be accountable for all the aspects of the work (notably accuracy/integrity of data). All authors reviewed and contributed critically to the manuscript. All authors read and approved the final version.

## Funding

The authors have nothing to report.

## Ethics Statement

The authors have nothing to report.

## Consent

The patient signed an informed consent form concerning the publication of her data.

## Conflicts of Interest

Yves‐Marie Pers received speaking fees and/or honoraria for advisory board (less than $10 000 each) from Abbvie, Medac, UCB and Novartis. All remaining authors declare no conflicts of interest.

## Supporting information


**Table S1:** List of preferred terms excluded.

## Data Availability

Access to VigiBase is granted directly by the Uppsala Monitoring Centre (UMC) (https://who‐umc.org/). Each authorized user receives individual login credentials, which must not be shared. For transparency, the extracted data were compiled into an Excel file and can be made available upon reasonable request.
