# Supplementary material for: JAK Inhibitors and Memory Impairment: Disproportionality Analyses in the WHO Global Pharmacovigilance Database, VigiBase
Source: Fundam Clin Pharmacol. 2026 Jan 20;40(1):e70072. doi: 10.1111/fcp.70072 (PMC12819935; doi:10.1111/fcp.70072)
Supplement: Supplementary file 1 — Table S1: List of preferred terms excluded. [file FCP-40-0-s001.docx]

## SUPPLEMENTARY DATA

## Supplementary table. List of preferred terms excluded

| **Categories** | **Co-reported preferred terms (MedDRA)** | **Count** |
| --- | --- | --- |
| Vascular disorders | Cerebrovascular accident  Transient ischaemic attack  Cerebral haemorrhage  Intracranial aneurysm  Cerebral infarction  Cerebral small vessel ischaemic disease  Cerebral artery thrombosis  Cerebral ischaemia  Cerebellar ischaemia  Brain stem infarction  Basal ganglia infarction  Ischaemic cerebral infarction  Subarachnoid haemorrhage  Subdural haematoma  Vascular encephalopathy  Cerebral congestion | 110  19  7  6  3  2  1  1  1  1  1  1  1  1  1  1 |
| Degenerative disorders | Dementia  Mental impairment  Dementia Alzheimer's type  Cerebral atrophy  Demyelination  Neurodegenerative disorder | 48  42  22  3  2  1 |
| Brain disorders | Cerebral disorder  Nervous system disorder  Brain neoplasm  Encephalopathy  Posterior reversible encephalopathy syndrome  Meningitis  Hydrocephalus  Encephalitis herpes  Brain cancer metastatic  Progressive multifocal leukoencephalopathy  Meningitis viral  Meningitis herpes  Meningitis bacterial  Herpes zoster meningitis  Leukoencephalopathy  Colloid brain cyst  Glioblastoma multiforme  Intracranial mass | 23  12  7  5  2  2  2  2  2  2  1  1  1  1  1  1  1  1 |
| Brain injuries | Head injury  Concussion  Accident  Coma  Brain injury  Cerebrospinal fluid leakage  Brain operation  Craniocerebral injury  Brain contusion  Brain compression | 31  20  13  8  6  3  3  3  1  1 |
